# Neuroprotective Potential of Limonene and Limonene Containing Natural Products

**DOI:** 10.3390/molecules26154535

**Published:** 2021-07-27

**Authors:** Lujain Bader Eddin, Niraj Kumar Jha, M. F. Nagoor Meeran, Kavindra Kumar Kesari, Rami Beiram, Shreesh Ojha

**Affiliations:** 1 Department of Pharmacology and Therapeutics, College of Medicine and Health Sciences, United Arab Emirates University, P.O. Box 17666, Al Ain 17666, United Arab Emirates; 201970113@uaeu.ac.ae (L.B.E.); nagoormeeran1985@uaeu.ac.ae (M.F.N.M.); rbeiram@uaeu.ac.ae (R.B.); 2 Department of Biotechnology, School of Engineering & Technology, Sharda University, Greater Noida, Uttar Pradesh 201310, India; niraj.jha@sharda.ac.in; 3 Department of Applied Physics, School of Science, Aalto University, 00076 Espoo, Finland; kavindra.kesari@aalto.fi; 4 Department of Bioproducts and Biosystems, School of Chemical Engineering, Aalto University, 00076 Espoo, Finland

**Keywords:** limonene, citrus terpenes, neuroprotection, neuroprotective, neurodegeneration, neurotherapeutics

## Abstract

Limonene is a monoterpene confined to the family of *Rutaceae*, showing several biological properties such as antioxidant, anti-inflammatory, anticancer, antinociceptive and gastroprotective characteristics. Recently, there is notable interest in investigating the pharmacological effects of limonene in various chronic diseases due to its mitigating effect on oxidative stress and inflammation and regulating apoptotic cell death. There are several available studies demonstrating the neuroprotective role of limonene in neurodegenerative diseases, including Alzheimer’s disease, multiple sclerosis, epilepsy, anxiety, and stroke. The high abundance of limonene in nature, its safety profile, and various mechanisms of action make this monoterpene a favorable molecule to be developed as a nutraceutical for preventive purposes and as an alternative agent or adjuvant to modern therapeutic drugs in curbing the onset and progression of neurodegenerative diseases. This manuscript presents a comprehensive review of the available scientific literature discussing the pharmacological activities of limonene or plant products containing limonene which attribute to the protective and therapeutic ability in neurodegenerative disorders. This review has been compiled based on the existing published articles confined to limonene or limonene-containing natural products investigated for their neurotherapeutic or neuroprotective potential. All the articles available in English or the abstract in English were extracted from different databases that offer an access to diverse journals. These databases are PubMed, Scopus, Google Scholar, and Science Direct. Collectively, this review emphasizes the neuroprotective potential of limonene against neurodegenerative and other neuroinflammatory diseases. The available data are indicative of the nutritional use of products containing limonene and the pharmacological actions and mechanisms of limonene and may direct future preclinical and clinical studies for the development of limonene as an alternative or complementary phytomedicine. The pharmacophore can also provide a blueprint for further drug discovery using numerous drug discovery tools.

## 1. Introduction

Phytochemicals continue to garner intense attention in the pharmaceutical community as well as the scientific fraternity due to their countless bioactivities. Nowadays, many pharmaceutical drugs are derived from natural plants and herbs [1]. Isoprenoids, which are isoprene derivatives also known as monoterpenes, have shown a powerful potential in phytotherapy [2]. Apart from being a defensive tool in plants for expelling pests, serving as signaling hormones, attracting insects for herbivore control, pollination, and seed spreading, monoterpenes play a valuable role in combating diseases through protecting against the deleterious effects of aberrant biological processes [3]. Monoterpenes derived from a number of therapeutic plants are being used as a conventional medicine for treating diabetes, hypertension, and inflammation [4,5,6].

Among terpenes, limonene is a monocyclic monoterpene found abundantly in citrus fruit peel oils [7]. Limonene’s structure has a chiral center, and it is an optically active compound with two existing enantiomers, (*R*)- and (*S*)-limonene. Due to the presence of two double bonds and a six-membered ring in the structure, it is found as D (or R)- and L (or S) isomers or as a mixture [8]. In stereochemistry, (+) enantiomers, also called dextrorotatory, abbreviated D, rotate plane-polarized light clockwise and (-) enantiomers, also called levorotatory, abbreviated as L, rotate it counter-clockwise. The D-isomer is also known as (R) configuration and L-isomer is often known as (*S)* configuration. However, D and L denote relative configuration, whereas R and S denote absolute configuration. D-limonene is considered the main abundant compound in citrus peels and the most active, where the majority of the biological activities are attributed to it [9]. The R-isomer possesses the characteristic smell of orange, whereas S-isomer is characterized by a lemon flavor and racemic limonene, known as dipentene. The S-enantiomer is also known as L-limonene or (-)-limonene with the CAS number 5989-54-8 and EINECS number 227-815-6. Limonene occurs naturally as the R-enantiomer or D-limonene or (+)-limonene, with the CAS number 5989-27-5, and EINECS number 227-813-5. D-limonene is a very stable compound and upon heat treatment, it can be racemized to dipentene.

The essential oils (EOs) from oranges mainly consist of (*R*)-limonene (95%), while the lemon peel consists mainly of (*S*)-limonene. Besides, D-limonene is a valuable raw material for the production of a variety of compounds as well as the synthesis of polymers, as it can undergo isomerization, epoxidation, addition and hydrogenation [10]. It is often employed as a substrate for the generation of numerous oxy functional derivatives following microbial transformation or chemical synthesis. It is a very cheap starting material and a widely available and accessible byproduct to produce many other flavor compounds.

Limonene can be extracted from more than 300 EOs, principally from *Citrus spp.* where it can reach up to 98% of the *Citrus spp.* oil’s content [11]. The genus Citrus (*Rutaceae*) is the family of citrus which includes the most planted fruits rich in limonene, such as orange, lemon, and mandarin [12]. The EOs of *Rutaceae* citrus contain varying amounts of limonene, such as bitter orange (*Citrus aurantium*) (67.90–90.95%), lemon (*Citrus limon*) (37.63–69.71%), orange maltaise (*Citrus sinensis*) (81.52–86.43%), and mandarin (*Citrus reticulate*) (51.81–69.00%) [13]. The percentage occurrence of limonene in EOs of plants and plant products are provided in Table 1.

Limonene is a resulting byproduct of the citrus industry, where it is generated by condensation through steam evaporators during the synthesis of citrus molasses. Besides, it can be produced by the steam distillation of citrus peels and pulp or by the deterpenation of citrus oils [14]. The majority of studies employed D-limonene, and it is commonly reported in the herbal medicinal preparation intended for experimental studies, bioanalytical studies and human studies. The stereochemistry or chirality is an important aspect in drug development as the specific isomer may have different pharmacological action and can be specific in nature to cause beneficial or adverse effects. Likewise, one of herbal medicinal products, silymarin, consisting of optically pure components, showed the crucial role of stereochemistry in inhibiting the aggregation of amyloid-beta (Aβ), a pathogenic hallmark of Alzheimer’s disease [15]. Studies have revealed that D-limonene, which is isolated from medicinal plants, has a number of therapeutic effects, including anti-cancerous [16], anti-nociceptive [17], gastroprotective and healing of gastric ulcer [18], antioxidant [19] and anti-inflammatory [20] properties, and effectiveness in improving lung function [21].

Neurodegenerative diseases (NDs) are a diverse group of disorders that occur by the degradation and subsequent loss of neurons. These changes in the human brain can lead to the cognitive or functional deterioration of the patient with time [22]. Its pathological condition characterizes many neurodegenerative diseases, including Alzheimer’s disease (AD), Parkinson’s disease (PD), and multiple sclerosis (MS). NDs share many fundamental processes associated with progressive neuronal dysfunction and death, such as oxidative stress, abnormal protein deposition, damaged mitochondrial function, the induction of apoptosis, impairment of proteostasis, and neuroinflammation [23].

Phytochemicals are shown to have a protective action against oxidative stress and neuroinflammation, which are the major hallmarks of NDs [24]. A great number of terpenes or terpene derivatives from natural sources have been described to have antioxidant and anti-inflammatory bioactivities [25]. Multivariate data analysis revealed a good correlation between some monoterpenes, e.g., limonene, with the antioxidant capacity of the natural extract [26].

Recent evidence indicates that limonene in particular has a beneficial effect in NDs due to its antioxidant and anti-inflammatory potentiality and may be used for treating or preventing NDs. This review has been compiled based on a review of the existing published articles confined to limonene or limonene-containing natural products investigated for their neurotherapeutic or neuroprotective potential. A wide variety of keywords were used, including limonene AND/OR, neuroprotective, neurotoxicity, neurodegeneration, Alzheimer’s disease, multiple sclerosis, Parkinson’s disease, epilepsy, anxiety, depression, encephalitis, Huntington disease, cerebral ischemia, stroke, cerebrovascular disease, neuroprotection, neurological diseases. All the articles available in English or the abstract available in English were extracted from different databases that offer access to diverse journals. These databases include PubMed, Scopus, Google Scholar, and Science Direct. There was no set time limit for studies included for reviewing in this paper. The available in vitro and in vivo studies demonstrating the pharmacological mechanisms and actions of limonene in NDs are presented in synoptic tables and figures in disease specific sections.

This review sheds light on the key role of limonene and limonene-containing phytochemicals that have shown promise as potential neuroprotective agents in NDs.

## 2. Neuroprotective Effects and Mechanisms of Limonene

### 2.1. Alzheimer’s Disease

Alzheimer’s disease (AD) is one of the prevalent neurodegenerative diseases that is mainly featured by cognitive impairment and gradual memory regression driven by the regression in cholinergic neurotransmitter; acetylcholine (Ach) levels and the accumulation of Aβ and neurofibrillary tangles (NFTs) [27]. Subsequently, augmenting the levels of Ach as well as preventing the proteinaceous deposits aggregation have become the cornerstone for alleviating AD associated symptoms. The intrinsic mechanism for this is targeting and inhibiting the two enzymes responsible for Ach degradation, which are acetylcholinesterase (AChE) and butyrylcholinesterase (BChE).

Accordingly, limonene was investigated for its activity against AChE and BChE. Limonene showed anti-AChE and anti-BChE activity by 10 and 12% respectively [28]. Amyloid plaques formed as a result of the accumulation of Aβ, which is a pathological characteristic of AD that leads to impaired synaptic plasticity and cognitive function [29]. A recent study investigated the efficacy of limonene against Aβ42-induced neurotoxicity in drosophila, a fruit fly model of AD. The study showed that limonene repressed the neuronal cell death induced by Aβ42 and decreased the reactive oxygen species (ROS) levels that had a negative impact on ERK phosphorylation. It was concluded that limonene was not a direct inhibitor of ERK, but its antioxidant property prevented ERK activation. The rough eye phenotype (REP) caused by Aβ42 can show the extent of neurotoxicity and by which AD can be observed [30]. REP investigation indicated that flies fed with limonene had relieved REP in a comparable manner to donepezil that was used as the positive control, as it was also effective in alleviating REP. The anti-inflammatory property of limonene was also tested to show a remarkable decrease in the activated glial cell number and nitric oxide (NO) expression in limonene-treated flies’ heads [31].

The neuroprotective role of *Citrus sinensis* (orange) byproducts was assessed by in vitro biological assays testing anti-inflammatory and antioxidant roles. It showed that monoterpenes such as limonene’s antioxidant potentiality is strongly correlated with their neuroprotection [32].

Furthermore, the efficiency of *Citrus medica L.* that contains limonene as the most abundant constituent (15.20%) was found to have anti-AChE activity. This activity was associated with the presence of a hydrocarbon skeleton in limonene, which is considered as a hydrophobic ligand that can contribute to the interaction with the hydrophobic active site of AChE. The same study confirmed the antioxidant capacity of the plant based on in vitro assays [33].

Limonene also had a beneficial effect on scopolamine-induced amnesia, where it improved the alterations caused by scopolamine in short-term memory that was observed in multiple cognitive ability scrutinizing tests. This is consistent with an attenuation of AChE activity that was increased in the brains of rats treated with scopolamine. The microtiter assay revealed an inhibition of 24.97% and 69.12% for AChE and BChE respectively. This is in addition to the emphasis on limonene’s capability to reduce oxidative stress biomarkers such as protein carbonyls, and malondialdehyde (MDA), and to increase superoxide dismutase (SOD), catalase and glutathione (GSH) [34].

As a supportive evidence, Zhou et al. examined how limonene affects monoamine neurotransmitters in both the striatum and hippocampus which both have an essential role in memory processing. Therefore, dopamine concentration was found to be substantially lower in the scopolamine-administered group compared to the limonene group. In contrast, DOPAC concentration; the metabolite of dopamine, was higher in the scopolamine group than in limonene group [35]. Moreover, the inhalation of *T. articulata* containing 7.34% limonene subsided the memory errors occurred in Aβ_1-42_-administered groups that were reported in multiple testing mazes [36].

In addition, *Aloysia citrodora*, which composes up to 20.1% limonene, exhibited neuroprotection against β-amyloid-triggered neurotoxicity [37]. The AChE inhibitory activity by microplate assay revealed that *black pepper* oil possesses a potent anti-AChE activity as well as β-amyloid aggregation inhibitory activity. At the same time, limonene possessed a strong inhibitory activity (IC_50_ value of 3.77 μg/mL), based on which limonene as an active compound is believed to be responsible for *black pepper* oil activity in this assay [38].

**Figure 1:**
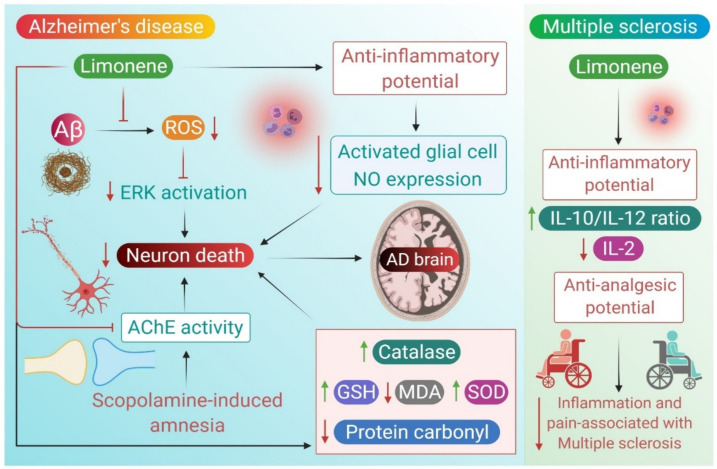
Neuroprotective mechanisms of limonene in Alzheimer’s disease and multiple sclerosis.

Altogether, this elucidates how limonene plays a vital role in the pathological cascade of AD, and how this therapeutic role can be beneficially exploited in targeting AD-related dysfunction, whether by its antioxidant or neuroprotective ability. The pharmacological mechanisms and effects of limonene in AD are shown in Figure 1.

### 2.2. Multiple Sclerosis

Multiple sclerosis (MS) is one of the debilitating neurodegenerative diseases, mediated by an autoimmune reaction involving the invasion of immune cells to the central nervous system (CNS). The pathological hallmarks of MS consist of inflammation, demyelination, reactive gliosis, and axonal degeneration [39]. To date, MS is known to be triggered by microbial pathogens, hence it is essential to develop antibiotic agents that have the ability to inhibit the microbial activity, thus enhancing disease recovery and reducing severity.

*Cakile maritima* solvent extracts displayed good 1,1-diphenyl-1-picrylhydrazyl (DPPH) radical scavenging and bacterial growth inhibitory activity against most bacterial species, including *Proteus mirabilis, Proteus vulgaris* and *Pseudomonas aeruginosa* [40]. *Terminalia sericea* extract was investigated for its ability to prevent and treat MS by blocking the trigger of the disease’s etiology. The extract of *Terminalia sericea* inhibited *Acinetobacter baylyi* and *Pseudomonas aeruginosa* growth involved in MS induction, with high and low potency respectively [41].

The cannabinoid receptor type 2 (CB2) was reported to contribute to analgesia by blocking the release of inflammatory mediators by cells located surrounding the nociceptive nerve terminals. In addition, the activation of peripheral CB2 receptors suppresses pain signal transmission into the CNS. Based on the fact that CB2 receptors are expressed in several types of inflammatory cells, it is reasonable to anticipate that the activation of peripheral CB2 receptors may have an analgesic effect in conditions of inflammatory hyperalgesia and neuropathic pain such as MS [42].

The D-limonene structure exists as component of cannabibidiol structure, with no resemblance to the structure of tetrahydrocannabinol. Terpenes are known to enhance the activity of cannabinoids in synergy. In recent years, cannabinoid–cannabinoid or terpenoid–cannabinoid interactions have been believed to exert the “entourage effect”, which was first described in cannabis by Ben-Shabat and colleagues. The “entourage effect”, which is presumed to be observed following synergistic interaction between terpenes and cannabinoids, is considered to positively contribute to the effect of cannabinoids [43]. Based on this, it has been reasonably speculated that limonene is one of the cannabis terpenoids that activates the CB2 receptor, resulting in an analgesic effect [44].

In an in vitro study, it was found that limonene has an efficient ability to increase the IL-10/IL-2 ratio and correspondingly enhance amounts of IL-10, which is a cytokine synthesis inhibitory factor that has a role as an anti-inflammatory element that inhibits proinflammatory Th1 cytokine production (IL-2) [45]. Relating to this, another study assessed the anti-inflammatory and antinociceptive potentiality of D-limonene epoxide. The anti-inflammatory potential was evaluated using agents to induce paw edema and the antinociceptive effect was evaluated by a writhing test. Animals received D-limonene epoxide had reduced paw edema and reduced abdominal contortions induced by acetic acid and paw licking time. D-limonene epoxide prevented the release of inflammatory mediators, inhibited the vascular permeability, reduced the migration of neutrophils, and displayed systemic and peripheral analgesic effects dependent on the opioid system [46].

These observations cumulatively support the anti-inflammatory, and analgesic potential of limonene and are correspondingly suggestive of therapeutic uses against inflammation and pain associated with MS. The pharmacological mechanism of limonene in relieving MS-associated symptoms is depicted in Figure 1.

The available in vitro and in vivo studies demonstrating the pharmacological mechanisms and actions of limonene in AD and MS are presented in Table 2 and Table 3, respectively.

### 2.3. Migraine

Migraine is a neurological disorder that is characterized by recurrent excruciating throbbing headaches. It is usually associated with nausea, phonophobia, and photophobia and sensory symptoms that can be provoked by lifestyle-related factors. The pathogenesis of migraines is known to be dependent on the activation of the trigeminovascular pain pathway [47]. Therapeutic interventions for migraine are imperatively preventative, targeting the pain pathway. There have been several studies that aim to discover an unmet number of therapeutic agents that would fit as preventative therapy against migraine.

A randomized placebo-controlled pilot clinical trial assessed the effects of *Anise* (*Pimpinella anisum* L.) EOs, and showed that the *Anise*-treated group showed a decrease in the frequency, mean duration, the severity of migraine attacks and analgesic consumption as well, compared to the placebo group [48]. Another longitudinal prospective phase 2 non-controlled cohort study evaluated the effects of *Lippia alba* (Mill.) N. E. Brown; a plant with many effects on the CNS, on pain frequency and the intensity in migraine patients. It was demonstrated that the group who received *Lippia alba* had a lower pain intensity with lesser frequent symptoms [49].

**Figure 2:**
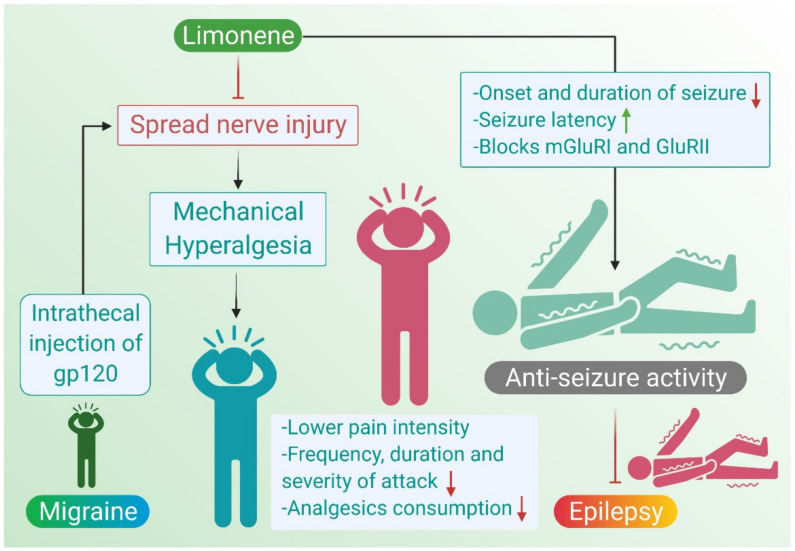
Neuroprotective mechanisms of limonene in migraine and epilepsy.

A conducted survey revealed that most patients preferred medicinal cannabis. This would in turn pinpoint the robust analgesic, anti-inflammatory, and anti-emetic properties of cannabis. Besides, patient tend to replace their prescribed analgesics, among which are opioids, with medicinal cannabis, conferring cannabinoids an opioid-sparing effect [50]. Furthermore, it was illustrated that limonene could inhibit the spared nerve injury (SNI)-induced mechanical hyperalgesia in mice that received intrathecal injection of glycoprotein (gp120) [17]. These data reflect the ability of limonene to mitigate migraine by reducing hyperalgesia associated with it. The possible benefits and actions of limonene in migraines are depicted in Figure 2.

### 2.4. Epilepsy

Epilepsy is characterized by the predisposition to develop re-occurring seizures, due to either the hyper excitability of excitable neurons represented by glutamatergic neurons or the low responsiveness of inhibitory GABAergic neurons in the cerebral cortex [51]. An epileptic seizure is defined conceptually as a transient occurrence of signs and symptoms due to abnormal excessive or synchronous neuronal activity in the brain [52]. Currently available antiepileptic drugs do not affect epileptogenesis and are associated with serious side effects, including teratogenicity, chronic toxicity and adverse effects on cognition and behavior [53]. There is an urgent need to find newer antiepileptic agents with better safety and efficacy profiles.

Several phytochemicals attracted great attention by multiple studies for their advantageous use as antiepileptic agents. In maximal electroshock (MES) and pentylenetetrazole (PTZ)-induced epilepsy in mice, *Artemisia dracunculus L*. EOs exerted an anticonvulsant activity in a dose-dependent manner. The observed anticonvulsant and sedative effects were related to the presence of monoterpenoids in it [54].

The anticonvulsant activity of the EOs of *Citrus aurantium* was assessed in PTZ- and MES-induced convulsions in mice. *C. aurantium* essential oil prevented PTZ-induced clonic seizures by 92.1%, and 100% against death resulting from electric shocks. The same study reported that the protective effect of *C. aurantium* was abolished after the administration of flumazenil, a competitive GABA_A_ benzodiazepines receptor antagonist, revealing the fact of the involvement of γ-aminobutyric acid (GABA) transmission in the therapeutic effect of *C. aurantium* [55].

As an additional support, Abbasnejad et al. reported consistent results, where *C. aurantium* extract lowered the onset and duration of seizure and protected against mortality resulting from seizures [56]. In a PTZ model of seizures in zebrafish, *C. aurantium* extract increased seizure latency by 119% compared to controls. Gabazine; a GABA_A_ receptor antagonist, was used to decide whether the improvement in seizure latency caused by the *C. aurantium* extract was dependent on an interaction with GABA_A_ receptor. The changes in seizure latency caused by gabazine co-administered with *C. aurantium* were not significant, excluding GABA_A_ receptor as a mediator of the effect of *C. aurantium* extracts. The extract also reduced [^3^H] Glu binding referring to an interaction with glutamate receptors, with particularly NMDA receptors; mGluRII and mGluRI, providing a proof of the anticonvulsant properties of *C. aurantium* and a mechanism involving NMDA and mGluRI and II receptors [57].

The intraperitoneal administration of limonene increased the latency in convulsions as well as the survival rate in animal with induced seizures. However, it was found that the oral administration of limonene has the same effect, only after doubling the dose. The concomitant administration of limonene with diazepam (DZP) showed a notable potentiation of its effect, suggesting a similar mechanism of action. As limonene is a constituent of the essential oils of *Lippia alpa* it can be said that *Lippia alpa* possesses an antiepileptic activity [58].

*Cinnamosma madagascariensis Danguy* EOs were also tested for their anticonvulsant ability. Its essential oils restrained convulsions induced by PTZ with moderate sedative effects [59]. *Piper guineense* EOs prevented episodes of convulsion and offered 100% protection against the PTZ-induced convulsions, similar to diazepam [60]. The effect of EOs extracted from the root of *Angelica archangelica L*. was investigated in both electrically and chemically induced seizures. The EOs decreased the duration of tonic convulsions, enhanced the recovery in maximal electroshock-induced seizures, delayed the time of onset of clonic convulsions, and protected against mortality in PTZ-induced seizures [61].

On the other hand, semicarbazones are believed to be the pharmacophores that are responsible for the anticonvulsant activity of most anti-epileptic drugs [62]. Several compounds were used to synthesize semicarbazones resembling analogues, among which is limonene, that was used as a prominent moiety for its ability to cross the blood–brain barrier. Analogues with limonene ring demonstrated robust anticonvulsant activity due to the closed ring in limonene, conferring it a preferable conformation to fit on the corresponding receptor binding site, compared with other compound-based semicarbazones [63].

These studies conclude that plant-based extracts exhibit significant anti-seizure activity against chemically and electrically induced seizures, and the same can be attributed to monoterpenes, especially limonene, present in them. The antiepileptic actions of limonene are depicted in Figure 2. The available in vivo studies demonstrating the pharmacological mechanisms and actions of limonene in epilepsy are presented in Table 3.

### 2.5. Anxiety

Anxiety disorders are defined as being abnormally aroused which can emerge from many triggering causes. It also encompasses the concept of over exaggerated tension and the fear of uncertain and unknown upcoming life events [64]. The etiology of anxiety has been linked to both defects in the brain function and environment-implied stresses [65]. Neurotransmitters also form a part of anxiety etiology, where serotonin, norepinephrine and gamma-aminobutyric acid pathway dysregulation is critical in modulating the physiological response inducing anxiety [66,67]. Current available pharmacotherapy for anxiety has been associated with troublesome side effects, rendering finding alternative therapeutic agents with less comparable drawbacks necessary [68].

GABA neurotransmission is considered as the cornerstone modulator of epilepsy pathology, considering the GABAergic pathway as the main inhibitory pathway in the brain. Benzodiazepines are the drug class of choice for attenuating anxiety, by activating GABA_A_ receptor and mediating an inhibitory state [69]. The effect of *Citrus aurantium L*. EOs was examined on anxiety and its correlation with the GABAergic pathway. The anxiety-related behavior of mice was evaluated by the elevated plus maze test (EPM). The study results concluded that treatment with *C. aurantium* EOs significantly increased the time spent in the open arms as well as the number of entries into them [70].

To examine the participation of the GABAergic system in mediating the anxiolytic effect of *C. aurantium*, diazepam co-administration with *C. aurantium* was found to enhance the resulting anxiolytic effect, suggesting an involvement of the GABAergic system [71]. To better identify the activity of *C. aurantium* EOs, it was assessed in a light–dark box experimental model. Mice treated with *C. aurantium* increased the time spent by mice in the light chamber and the number of transitions between the two compartments [72].

A previous study stated that limonene can interact with GABA_A_ receptor, producing an anxiolytic effect by increasing GABA concentration in the brain [73]. This in turn explains the anxiolytic effect of *C. aurantium* due to its limonene constituent. Another study comprehensively evaluated the ability of preparations obtained from *C. aurantium* to induce anxiolytic and improving the hypnotic effects in an experimental mice model by measuring the sleeping time induced by sodium pentobarbital and the EPM test. Extracted EOs increased the sleeping time induced by barbiturates, improving the hypnotic effect of pentobarbital as well as the time spent in the open arms of the EPM, indicating an anxiolytic effect [74].

In a trial to discover the underlying mechanism of *C. aurantium* EOs, a group of mice were treated with WAY100635, a selective 5-HT_1A_ receptor antagonist, which led to the abolishment of the anxiolytic effects of EOs. Therefore, it can be inferred that the serotonergic system is involved in mediating the anxiety attenuating effects of *C. aurantium*. However, the same paper scrutinized the effect of the co-administration of flumazenil with *C. aurantium* EOs and found out that the anxiolytic effect of the EOs was not impacted by this concurrent administration. Consequently, this finding suggested that EOs’ function is independent from the GABA–benzodiazepine receptor complex [75].

Citrus fragrances have been used by aroma therapists to treating anxiety symptoms. Based on this, *Citrus latifolia* and *Citrus reticulata* EOs were examined for their activity against anxiety. The anxiolytic activity was manifested by the prolonged time spent in a light compartment in a light–dark box test and the decreased marbles buried in the marble burying test [76]. *Citrus limon*, *Citrus sinensis* (sweet orange) and *Foeniculum vulgare* EOs were shown to increase the time spent in open arms and decrease time in closed arms in the EPM test [77,78,79].

Other studies were carried out to examine the anxiety attenuating abilities of other phytochemicals. The inhalation of *Bergamot* EOs attenuated the activity of the hypothalamic–pituitary–adrenal (HPA) axis by decreasing the secreted corticosterone levels after stress due to exposure to the EPM test [80]. To elucidate the possible mechanism behind this anxiolytic effect, a previous study concluded that *Bergamot* EOs resulted in a significant rise in the GABA expression in the hippocampus of rats [81]. At the same time, blockade of the GABAergic pathway by a GABA_A_ receptor antagonist showed, in turn, an increased corticotropin-releasing factor (CRF) release, reflecting the involvement of GABA in the regulation of HPA activity, depending on the morphological fact that GABA neurons terminate at CRF neurons [82]. In another words, it can be said that *Bergamot* oils exert their anxiolytic effect by the GABA-suppressed HPA axis.

Regarding the role of limonene as a part of *Bergamot* EOs, Zhou et al. demonstrated the ability of limonene to increase GABA levels in the brain of rats, which forms a supportive evidence for the GABA-dependent mechanism [83]. *Bergamot* essential oils (BEOs) were also examined for their anxiolytic effects by using an open field task (OFT), EPM, and a forced swimming task (FST) in rats. This study further compared the impact of BEOs to that of diazepam on behavioral tests. The results indicated that BEOs have anxiolytic effects observed by animal behavioral tasks [84].

EOs are excessively used in aromatherapy in mild mood disorders and to mitigate symptoms of anxiety triggered by stress [85]. Studies investigated the effect of the odor of EOs on anxiety resulting from undergoing medical procedures. A prospective study indicated that exposure to the ambient odor of orange or *Citrus sinensis* has a relaxant effect, reflected in a more elated mood and a higher level of tranquility in patients awaiting dental treatment [86]. A second finding supported the previous study that odors are able to improve emotional states, where patients who inhaled orange and lavender odors showed lower anxiety and improved mood [87]. This indicates that the usage of odors is advantageous in reducing anxiety in patients undergoing dental procedures.

A phase 2, randomized, placebo-controlled clinical trial demonstrated that the use of powdered leaves of *Aloysia polystachya* in anxiety cases was superior to the placebo in decreasing the Hamilton Anxiety Ranking Scale (HAM-A) [88]. The essential oil of *Lippia alba* was examined for the ability to reduce anxiety levels. The anxiety index was evaluated pre- and post-test by State-Trait Anxiety Inventory. The outcome was a decline in the post-test phase in comparison with pre-test in the group treated with aromatherapy contained *Lippia alba* [89].

Another study evaluated the possible anxiolytic effect of sweet orange (*Citrus sinensis*) aroma in healthy participants who were subjected to an anxiogenic situation. The individuals who inhaled test aroma had no significant perturbation in state anxiety, subjective tension, and tranquility levels during the anxiogenic situation, indicating an anxiolytic activity of sweet orange EOs [90].

*Lavandula Angustifolia Mill.* EOs’ anxiolytic effect on preoperative anxiety in patients undergoing diagnostic curettage was evaluated by Spielberger’s questionnaire. The antioxidant activity of the EOs was confirmed by DPPH assay and had a good DPPH radical-scavenging potential. Clinical results delineated that the average anxiety score was lower in the case group than the control. The study demonstrated a positive relationship between EOs’ components and their antioxidant activity in decreasing anxiety in patients going for curettage, especially the night before the surgery [91].

An experimental study investigated whether the EOs of *Lavandin* are more effective than standard care in reducing preoperative anxiety. The *Lavandin* group had significantly lower anxiety on a visual analog scale, which was used to assess anxiety on admission, suggesting that the use of *Lavandin* is a simple, low-risk, cost-effective intervention with the potential to improve preoperative outcomes and increase patient satisfaction [92].

Stress stimulates a hormonal response along the hypothalamus–pituitary–adrenal (HPA) axis, which can impair the ortho/parasympathetic balance critical for a harmonious life and leads to hormonal imbalance [93]. D-Limonene was proved to provide an anti-stress action by disrupting ortho/parasympathetic parameters, as well as central neurotransmitter functionality. A functional observational battery was used on rats exposed to non-pathological stress. The spontaneous locomotor activity and emotional state (anxiety) were assessed by counting de-ambulation in an open arm field. Significant differences were observed for behavioral effects such as irritability, which was remarkably less after D-limonene or perillyl alcohol (POH) ingestion compared to the vehicle group. Similarly, D-limonene-treated rats reacted less to toe pinch and with less fear and startling reaction upon rattling. This study shows that D-limonene exhibits through its metabolite POH a remarkable anti-stress activity, reflecting the role of the endogenous metabolization of the terpene [94].

Corticosterone (a stress-induced hormone) elicits oxidative stress and an inflammatory process ending in cell apoptosis and neurological changes. AMP-activated protein kinase (AMPK), a serine/threonine protein kinase, mediates oxidative stress and inflammatory response by suppressing NF-κB nuclear translocation [95]. The suppression of NF-κB translocation into the nucleus inhibits the transcription of target genes, including genes involved in encoding for proinflammatory cytokines [96].

A recent study assessed the anticipated neuroprotective effect of D-limonene on a corticosterone-induced neurotoxicity model in PC12 cells. D-Limonene opposed the effects of corticosterone, stimulated AMPKα and inhibited NF-κB nuclear translocation through the upregulation of Silent mating type information regulation 2 homolog-1 (SIRT1). The administration of compound C, an AMPK inhibitor, intensively abolished the neuroprotective effects of D-limonene. In addition, limonene downregulated the levels of (MDA), nitric oxide (NO), NADPH oxidase, the expression of inducible nitric oxide synthase (iNOS), cyclooxygenase-2 (COX-2), interleukin 6 (IL-6), interleukin-1β (IL-1β), and tumor necrosis factor α (TNF-α), pro-apoptotic protein (Bax) and cleaved caspase-3. D-limonene upregulated levels of the antioxidant enzymes superoxide dismutase 1 (SOD1) and heme oxygenase 1 (HO-1) and the anti-apoptotic protein Bcl-2 while it reduced the number of TUNEL-positive cells. D-limonene has a neuroprotective effect mediated by the activation of the AMPKα signaling pathway, and hence inhibiting ROS and inflammatory factors [97].

Additionally, a study reported that limonene attenuates anxiety in the EPM model of anxiety in mice. The effects of limonene were reported at two concentrations of 0.5% and 1.0%, and showed that limonene improved all parameters assessed in the elevated plus maze test. To look into its mediating mechanism of action, flumazenil was used to block GABA_A_ receptor. The administration of flumazenil did not abolish the anxiolytic effect of inhaled limonene (1%), referring to a non-benzodiazepine-based anxiolytic activity of limonene [98].

Limonene also provided a notable rise in the open arm entries and permanence time in the EPM test, as compared with rats exposed to chronic immobilization and decreased stress-induced damage in CA1 area pyramidal neurons of the hippocampus [99]. Moreover, D-limonene epoxide (a mixture of cis and trans isomers) anxiolytic-like effects were investigated by the OFT and EPM test. D-limonene epoxide administered by intraperitoneal injection reduced the number of crossings, grooming and rearing in OFT. In the EPM test, D-limonene epoxide extended the time spent in and entrances to the open arms. However, flumazenil administration inverted these effects. This pinpoints an involvement of benzodiazepine-type receptors in facilitating the anxiolytic-like effects of D-limonene epoxide [100].

**Table 2:** The in vitro studies demonstrating the neuroprotective actions and mechanisms of limonene.

In Vitro				
Alzheimer’s Disease				
Phytochemicals	Dose	Model of Study	Mechanisms	References
Citrus medica L. cv. (Diamante citron)	164 μg/mL	In vitro based study	↓ DPPH ⇥ AchE	[33]
Aloysia citrodora Palau	0.001 and 0.01 mg/mL (w/v)	H_2_O_2_ (250, 200, 150, 100 and 50 μM) and β-Amyloid (2.5–25 μM)-induced neurotoxicity in CAD cells	↑ DPPH radical-scavenging Showed neuroprotection	[37]
Black pepper	4 mg/ml	β-amyloid (25 μM) in THP-1 cells	⇥ AChE ⇥ β-Amyloid aggregation ↓ COX-2	[38]
**Multiple Sclerosis**				
Cakile maritima Scop	10–80 μg/ml	Bacterial cultures of Proteus mirabilis, Klebsiella pneumonia, Proteus vulgaris and Pseudomonas aeruginosa	↓ DPPH radicals showed antimicrobial activity	[40]
Terminalia sericea	200 mg/ml	Bacterial cultures of Acinetobacter baylyi and Pseudomonas aeruginosa	(-)Acinetobacter baylyi and Pseudomonas aeruginosa	[41]
D-limonene	0.5–500 μM	Splenocytes culture	↑ IL-10/IL-2	[45]
**Anxiety**				
D-limonene	0.2, 0.4, 0.6, 0.8 and 1.2 μL/ml	Corticosterone (20, 50, 100, 200 and 400 μM) in PC12 cells	↑ phospho-AMPKα1/2 ↓ NF-κB nuclear translocation ↓ MDA and NO ↓ NADPH oxidase ↑SOD1 and HO-1 ↓ iNOS, COX-2, IL-6, IL-1β, and TNF-α, ↑ SIRT1 ↓ Bax and cleaved caspase-3, ↑ Bcl-2	[97]
D-limonene epoxide	0.9, 1.8, 3.6, 5.4 and 7.2 μg/ml	Griess method	↓ NO2^−,^ ↓ OH• ↓ TBARS	[101]

Symbol’s indications: ↑; increase, ↓;decrease, (-); reduce, ⇥; activity inhibition.

Another study attempted to investigate the anxiolytic activity of D-limonene epoxide (EL), with the aid of a marble burying test. Buried marbles by the group who received EL were less when compared with the diazepam and vehicle groups. The anxiolytic effect was reversed by pretreatment with flumazenil in the same pattern as it was with diazepam, an indication for the possibility that both EL and diazepam act similarly. The study also evaluated the antioxidant potential by in vitro and in vivo assays on adult mice hippocampus exposed to anxiogenic protocol. The in vitro antioxidant tests revealed a 50% inhibitory effect against the formation of nitrite ion, hydroxyl radical and reactive substances to thiobarbituric acid. The administration of EL mitigated the lipid peroxidation levels and nitrite content. This highlights the in vivo antioxidant role of EL, depending on its ability to decrease the formed oxygen and nitrogen reactive species. Furthermore, EL boosted the activity of antioxidant enzymes, including CAT and SOD in mice hippocampi [101].

An ethopharmacological study was conducted to examine the correlation between EOs components of *Alpinia zerumbet* and their anxiolytic effect. Anxiety-related behavior was examined by the light and dark box test (LD), OFT, and EPM tests. Behavioral tests showed that inhaling *Alpinia zerumbet* EOs had a positive anxiolytic effect, which was obvious in the increased time spent in the open arms in the EPM [102]. The inhalation of *Alpinia zerumbet* EOs increased motor activity simultaneously with the extension of the time in the open arms in the EPM [103].

**Figure 3:**
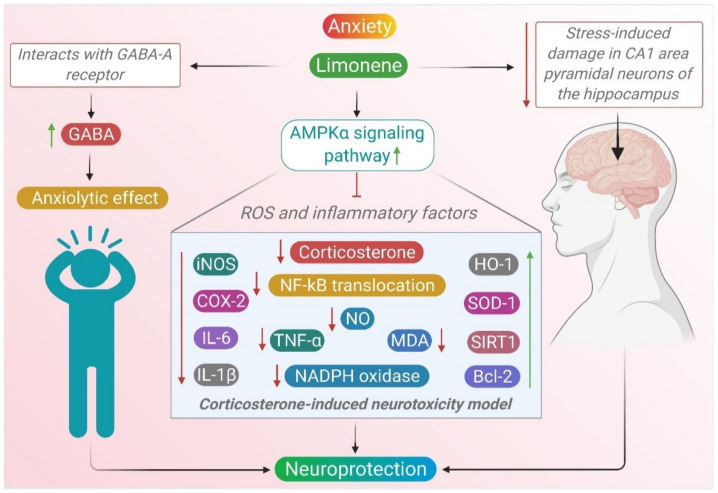
Neuroprotective mechanisms of limonene in anxiety.

This supports the traditional use of EOs containing limonene in altering emotional states. It also demonstrates that EOs used as ambient odors might be helpful to reduce anxiety and improve mood. A scheme depicted in Figure 3 summarizes the pharmacological mechanisms of limonene that mediate its anxiolytic effects. The available in vitro and in vivo studies demonstrating the pharmacological mechanisms and actions of limonene in anxiety are presented in Table 2 and Table 3, respectively.

### 2.6. Stroke

Stroke is defined as a clinical syndrome consisting of a rapid disturbance of cerebral function lasting more than 24 h. In some cases, stroke can lead to death, with no clear underlying cause other than a vascular origin. Stroke is categorized into two types: ischemic stroke and hemorrhagic stroke. Ischemic stroke occurs due to the occlusion of blood vessels, which obstructs the blood supply to the brain, whereas hemorrhagic stroke occurs due to rupture of blood vessels, leading to the spillage of blood in the intracranial cavity. Based on the specific region of brain on which the blood vessel has burst, the hemorrhagic stroke could be categorized as an intracerebral hemorrhage or subarachnoid hemorrhage. Ischemic stroke is more common, as up to 60–80% of all strokes are ischemic [104].

During brain ischemia, a complex vicious series of metabolic events are initiated, increasing the production of nitrogen and oxygen free radicals. These produced reactive species mediate the damage that occurs following transient brain ischemia [105]. Clot-busting thrombolytic treatment using recombinant tissue plasminogen activator (rt-PA) is the cornerstone of ischemic stroke therapy, but only reaches a few patients because of the timeframe constraint [106]. There is still limited data on the optimal management of intracerebral hemorrhage, but evidence favors the potential benefit of reducing the volume of clot [107].

Relying on one drug might not be adequate to preserve the ischemic neuroparenchyma, which has already been damaged due to inflammation, apoptosis, necrosis, the withdrawal of trophic factors, excitotoxicity, or the breakdown of membrane potentials. Consequently, a favorable therapeutic regimen should consist of therapeutic compounds capable of targeting different biological cascades. A huge number of experimental studies have been conducted in a trial to discover novel approaches that can preserve neuronal integrity and prevent neuronal death following ischemia [108].

The effectiveness of D-limonene in protecting against ischemia-associated cerebral injury was evaluated in stroke-prone spontaneously hypertensive rats (SHRsp). D-limonene was able to decrease the systolic blood pressure of SHRsp rats after stroke. Stroke triggering resulted in raised escape latency time, decreased time spent in the target quadrant in the probe trial, reduced ability to recognize between familiar objects and novel objects, and improved sensory neglect in the SHRsp rat. However, these symptoms were abolished by D-limonene.

D-limonene also prevented the increase in the cerebral infarct size in the SHRsp rats following stroke. D-limonene significantly reduced the mRNA expression of IL-1β, monocyte chemoattractant protein-1 (MCP-1) and COX-2 in SHRsp rats following stroke. The mRNA of vascular endothelial growth factor (VEGF) in the brain of SHRsp rats was upregulated by D-limonene. D-limonene also enhanced the activity of SOD and catalase, increased GSH content and reduced the MDA level, as well as dihydroethidium staining in SHRsp rats after stroke. The blockage of cerebral inflammation and vascular remodeling in addition to the antioxidant potentiality of D-limonene is thought to be responsible for its protective effects against ischemic damage in SHRsp rats [109].

Thrombosis occurring in the circulation outside the brain can lead to cerebral ischemia in most cases. Accordingly, the current therapeutic approaches strive to treat the resulting thrombotic pathology and reverse the underlying vascular defects. Following the occlusion of cerebral blood vessels, thrombolysis within a very narrow time window is by now the only approved and effective intervention. The effects of nanomaterials in *fennel (Foeniculum vulgare Miller*) seed were investigated on the cloth dissolution after spontaneously induced stroke in male mole rats (Spalax leucodon). The available data showed that both *fennel* constituents had a profound efficacy in dissolving the formed cloth [108].

Cerebral edema is a serious complication in patients with a developed stroke which can jeopardize a patient’s life. Until now, there has been no known effective treatment that has been discovered for cerebral edema [110]. The protective activity of *lavender* oil (*Lavandula angustifolia*) was evaluated on ischemic lesions and brain edema. Treatment with *lavender* oil could measurably reduce the infarct size, brain edema, and recover functionality after cerebral ischemia. *Lavender* oil was also efficient in reducing MDA level and increasing the activities of SOD, glutathione peroxidase, and total antioxidant capacity. Besides, it was observed that *lavender* oil increased vascular endothelial growth factor (VEGF) expression, but it could not downregulate the Bax-to-Bcl-2 ratio (pro-to anti-apoptotic proteins) in the rat brain [111].

One of the phenomena observed during reperfusion to recover the brain ischemia is the disruption to membrane lipids caused by lipolysis throughout ischemia and the oxidation to polyunsaturated fatty acids caused by radical-mediated peroxidation [112]. *Nigella sativa* oil (NSO) and *thymoquinone* (TQ), the active components of *Nigella sativa* seed oil, were reported for their potential against oxidative injury in many disease models [113]. The potential protective effect of TQ and NSO was explored by measuring the degree of lipid peroxidation following cerebral ischemia–reperfusion injury (IRI) in rat hippocampi. Pretreatment with TQ and NSO showed a decline in MDA levels as compared with ischemic rats [114].

Normally, brain tissues are less packed with antioxidant defense systems to counter ROS. Therefore, free radicals and ROS released by inflamed cells endanger the tissues surrounding the ischemic region. The blood–brain barrier (BBB) is known as being stringently impermeable due to the tight microvascular endothelial cells that surround the brain by their tight junctions and basal lamina. It is expected that damage to the endothelial basal layer starts 2 h after the onset of ischemia [115]. *Lavandula officinalis* was examined in an experimental ischemic model triggered by the transient blockage of the middle cerebral artery. *Lavandula officinalis* decreased the BBB permeability as well as MDA levels in both plasma and brain tissues of experimental rats compared with ischemic rats [116].

Cerebral ischemia can lead to reduced brain oxygen or cerebral hypoxia, resulting in death of the brain tissue. *Citrus aurantium* EOs were scrutinized on a rat’s hippocampus for their protective ability against consecutive transient cerebral ischemia and reperfusion injury. *C. aurantium* enhanced the spatial memory, passive avoidance learning, increased antioxidant defense, and attenuated lipid peroxidation resulting after ischemia [117].

Furthermore, phosphatidylinositol 3-kinase (PI3-K) targets Akt as one of its major downstream targets. Phosphorylated and activated Akt mediates cell survival by the resultant phosphorylation and inhibition of several proteins, including Bad, caspase-9, and GSK-3β. It has been elucidated that the activation of the PI3-K/Akt pathway may protect ischemic penumbra neurons from delayed cell death [118]. Interestingly, *Bergamot* essential oils (BEOs) were demonstrated to activate the PI3-K/Akt survival pathway in neurons deprived from trophic factors [119]. Hence, BEOs were investigated for their neuroprotective potential on a rat model of focal brain ischemia. BEOs increased the activation of the pro-survival kinase; Akt and enhanced the phosphorylation of the troublesome downstream kinase, GSK-3β, whose activity is negatively regulated via phosphorylation by Akt. BEOs also reduced the efflux of aspartate and glutamate, the major known excitatory amino acids in the frontoparietal cortex, generated after middle cerebral artery occlusion (MCAo), as well as robustly reducing infarct size after permanent MCAo [120].

**Figure 4:**
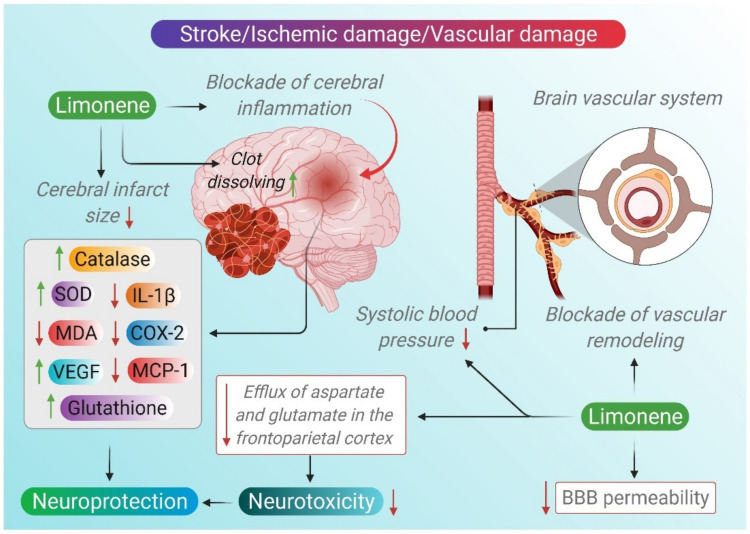
Neuroprotective mechanisms of limonene in cerebrovascular diseases.

Overall, plants rich in limonene can be possible therapeutic agents for treating stroke-associated cerebral and vascular damage. Figure 4 depicts the pharmacological actions of limonene in cerebrovascular diseases, including stroke. The available in vivo studies demonstrating the pharmacological mechanisms and actions of limonene in stroke are presented in Table 3.

### 2.7. Depression

Depression is a psychological disorder featured by sleeplessness, anorexia, anhedonia, sorrow, helplessness, guilt, and reduced functionality of the patient. Depression can also deteriorate the quality of life and increase the mortality rate due to suicidal deaths [121].

Available antidepressants are designed to target and restore monoamines imbalance. Studies found that current antidepressants are associated with serious adverse effects, and they are not equally effective with different individuals [122]. Thus, it is critical to discover new drugs with mood-normalizing abilities. Monoterpenes were found to possess mood and emotional improving effects by directly acting on the CNS and olfactory nerve [123].

Navel orange (*Citrus sinensis* (*L.*) *Osbeck*) essential oils (OEOs) and their main constituents, with the aid of the chronic unpredictable mild stress model (CUMS) created on mice, were investigated for their antidepressant effect. Limonene was estimated as the major constituent due to its high percentage of occupancy in the sniffing OEO environment. They found out that the limonene content in the brain of treated mice was higher than other compounds. They also suggested that limonene is responsible for the antidepressant effects of OEO because it delayed metabolism in mice brains. Finally, OEO inhalation attenuates depression-like behaviors induced by CUMS mice with corresponding decreased: body weight, sucrose preference, curiosity, mobility, as well as shortened immobile time tested by sucrose preference tests—SPTs—OFT, and FST.

To investigate the antidepressant mechanism of action of limonene, the study investigated the effect of limonene on neurotransmitter and HPA axis hormone levels in the hippocampus and prefrontal cortex. The supplementation of limonene restored the reduced levels of 5-HT, DA, and NE due to CUMS. Limonene also reduced CRF and corticosterone levels in the hippocampus and prefrontal cortex and reversed the decreased BNDF and Tr-κB in the hippocampus induced by CUMS. A similar effect was also reported after treatment with fluoxetine [124].

The antidepressant activities of the D-limonene from EOs of *S. terebinthifolius Raddi* fruit was investigated using spared nerve injury (SNI) as an experimental model for hyperalgesia and depression in rats. The oral administration of D-limonene reduced the SNI-induced increase in immobility in the FST [125]. The inhalation of EOs can regulate brain health and functions associated with mood and neurodegeneration, reflecting their bioavailability in brain.

**Figure 5:**
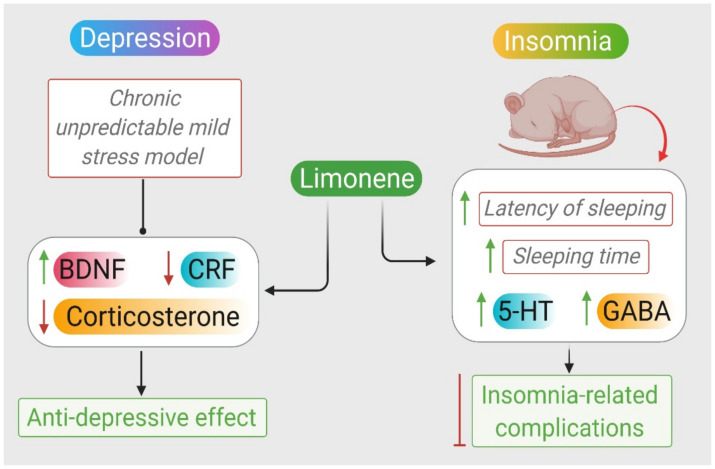
Neuroprotective mechanisms of limonene in depression and insomnia.

A study assessed the consequence of the inhalation of *Litsea cubeba (Lour.) Persoon* essential oils (LEOs) on mood states and salivary cortisol levels of healthy people. The inhalation of LEOs significantly improved the total mood disturbance and reduced the confusion among the healthy human subjects and reduced the salivary cortisol at a notable level [126]. This reasonably suggests that limonene might be a therapeutic compound against depression. Figure 5 illustrates the antidepressant effects of limonene. The available in vivo studies demonstrating the pharmacological mechanisms and actions of limonene in depression are presented in Table 3.

### 2.8. Insomnia

Insomnia is defined by the presence of a long sleep latency, frequent nocturnal awakenings, or prolonged periods of wakefulness during the sleep period, or even frequent transient arousals are taken as evidence of insomnia [127]. The most frequently prescribed drugs for the treatment of insomnia are benzodiazepine and z-drug groups such as zolpidem and zopiclone. The clinical pharmacological management requires awareness of their risk benefit profile when used to treat insomnia and the patients for whom they are indicated and contraindicated to minimizes the treatment risk–benefit ratio for each patient [128].

These agents exert this modulation by binding to a specific site on the GABA_A_ receptor complex, thereby changing the conformation of the receptor constituent proteins, which leads to an enhancement of the inhibition occurring when GABA binds to these receptors. This enhancement of inhibition is associated with a broad set of dose-dependent clinical effects, including sedation, anxiety reduction, seizure inhibition and myorelaxation [129].

**Table 3:** The in vivo studies showing the neuroprotective effects of limonene.

In Vivo				
Alzheimer’s Disease				
Phytochemical	Dose	Model of study	Mechanisms	Reference
Limonene	50 μg/mL or 200 μg/mL	Drosophila model	↓ rough eye phenotype ↑ survival rate, ↓ ROS ↓ ERK phosphorylation ↓ glial cells ↓ NO, ↓ Drs-GFP	[31]
Essential oil mixure	200 μL of 1% or 3%	Scopolamine (0.7 mg/kg b.w., i.p)-induced amnesia in rats	⇥ AChE and BChE ↑ GSH, ↓ SOD, CAT and MDA ↑ short-term memory	[34]
Limonene	100 mg/kg	Scopolamine (1 mg/kg)-induced dementia in rats	↑ memory ↓ DOPAC/dopamine ⇥ AChE	[35]
Tetraclinis articulata	200 μL of 1% and 3%	Aβ1-42 (4 μL i.c.v.)-induced Alzheimer’s disease amyloidosis	↓ DPPH and ABTS radicals ↑ alternation behavior in Y-maze ↓ memory errors ⇥ AChE ↑ SOD, CAT, GPX and GSH, ↓ protein carbonyl and MDA	[36]
**Multiple Sclerosis**				
Limonene epoxide	25, 50, 75 mg/kg	Carrageenan (500 μg/paw)-induced edema, acetic acid (10 mL/kg, i.p.) in Swiss mice	↓ paw edema ↓ leukocytes and neutrophils, MPO, IL-1β and TNF-α, ↓ Contortions number	[46]
**Migraine**				
Limonene	10 mg/kg orally	gp120-induced mechanical hyperalgesia in mice	↓ mechanical sensitivity ↓ IL-1β and IL-10 ↓ hyperalgesia ↑ SOD	[17]
**Epilepsy**				
Artemisia dracunculus L.	0.1, 0.15, 0.2, 0.4, 0.8 and 1.0 mL/kg	Electrical stimulus (50 mA), PTZ-induced seizures (60 mg/kg) in mice	(-)seizures	[54]
Citrus aurantium	5, 10, 20, and 40 mg/kg, i.p	PTZ (85 mg/kg)-induced convulsion in mice	(-)seizures ↓ mortality	[55]
Citrus aurantium	75, 150, 300 and 600 mg/kg, i.p	PTZ (90 mg/kg)-induced convulsion in rats	↓ onset of seizures ↓ duration of seizure ↓ mortality	[56]
Citrus aurantium	28 mg/ml	PTZ (3 mg/mL)-induced seizures in zebrafish	↑ latency of seizures	[57]
Limonene	200 mg/kg i.p	PTZ (80 mg/kg)-induced seizures in mice	↑ latency of seizures ↑ survival rate	[58]
Cinnamosma madagascariensis Danguy	0.4 and 0.8 mL/kg s.c	PTZ (60 mg/kg)-induced seizures in rats	(-)convulsions	[59]
Piper guineense	50–200 mg/kg i.p	PTZ (85 mg/kg)-induced seizures in mice	(-)convulsions	[60]
Angelica archangelica Linn.	50, 100, 200, 400, 500 mg/kg i.p	PTZ (80 mg/kg) and MES (50 mA)-induced seizures in mice	(-)clonic seizures	[61]
**Anxiety**				
Citrus aurantium L.	0.5%, 2.5%, and 5%, i.p	EPM test on mice	↑ time in open arms ↑ entries to open arms	[70]
Citrus aurantium L.	0.5%, 2.5%, and 5%, i.p. for 5 days	EPM test on mice	↑ time in open arms	[71]
Citrus aurantium L.	0.5 or 1.0 g/kg orally	Light–dark box test on mice	↑ time in light chamber	[72]
Citrus aurantium L.	0.5 or 1.0 g/kg orally	EPM on mice	↑ time in open arms	[74]
Citrus aurantium L.	1, 5 or 10 mg/kg/day orally	Light–dark box test on mice	↑ time in light chamber	[75]
Citrus latifolia and Citrus reticulate	0.5 to 2 g/kg orally	Light–dark box and marble burying tests on mice	↑ time in light chamber ↓ buried marbles	[76]
Citrus limon	50, 100 and 150 mg/kg orally	EPM test on mice	↑ time in open arms	[77]
Citrus sinensis	100, 200 or 400 μL for 5 min inhaled	EPM test on rats	↑ time in open arms	[78]
Foeniculum vulgare	50, 100, 200, 400 mg/kg orally	EPM, staircase and OFT on mice	↑ time in open arms ↓ rearing ↑ crossed squares	[79]
Bergamot	1.0%, 2.5% and 5.0% w/w inhaled	EPM and hole-board tests on mice	↑ entries to and time in open arms ↑ head dips ↓ corticosterone	[80]
Bergamot	100 μL/kg i.p	Measured on rats	↑ aspartate, glycine, taurine glutamate and GABA	[81]
(S)-Limonene	0,5,25,50 mg/kg orally	Foot shock (5 mA) induced stress in rats	↑ GABA ↓ glutamate ↓ corticosterone	[83]
Bergamot	100, 250, or 500 μL/kg i.p	OFT, EPM and FST on rats	↓ rearing, crossing and grooming ↑ entries to and time in open arms ↑ immobility and drowning recovery	[84]
D-Limonene	10 mg/kg orally	Functional observational battery on rats	↓ irritability and fear	[94]
D-Limonene	0.5%, 1.0% and 2.5%, inhaled	EPM on mice	↑ entries to and time in open arms	[98]
D-Limonene	10 mg/kg, orally	Plastic rodent restrainer for six hours induced stress on rats	↑ entries to and time in open arms	[99]
D-limonene epoxide	25, 50 and 75 mg/kg, i.p	OFT and EPM on mice	↓ crossing, rearing and grooming ↑ entries to and time in open arms	[100]
D-limonene epoxide	25, 50 and 75 mg/kg, orally	Marble burying test on mice	↓ buried glass beads ↓ lipid peroxidation ↓ NO_2_^−^ ↓ catalase, SOD	[101]
Alpinia zerumbet	8.7 ppm inhaled	LD, OF and EPM on mice	↑ entries to and time in open arms	[102]
Alpinia zerumbet	0.087, 0.87 or 8.7 ppm inhaled	Behavioral observation and EPM on mice	↑ locomotor activity ↑ entries to and time in open arms	[103]
**Stroke**				
D-limonene	20 mg/kg i.p	Transient middle cerebral artery occlusion for 60 min in rats	↓ BP ↓ escape latency time ↑ time in target quadrant ↑ capacity to distinguish novel objects ↑ grip strength ↓ sensory neglect ↓ IL-1β, MCP-1 and COX-2 ↑ VEGF ↑ SOD and CAT and GSH ↓ MDA and DHE staining	[109]
Lavender oil	100, 200 and 400 mg/kg i.p	Focal cerebral ischemia induced by transient occlusion of middle cerebral artery for 1 h in rats	↑ cerebral blood flow ↓ brain water content ↑ SOD, GSH-Px ↓ MDA ↑ TAC marker ↑ VEGF	[111]
Nigella sativa oil (NSO) and thymoquinone (TQ)	TQ: 2.5, 5 and 10 mg/kg NSO: 0.048, 0.192 and 0.384 mg/kg i.p	Transient global cerebral ischemia induced by four-vessel-occlusion for 20 min in rate	↓ MDA	[114]
Lavender	100 and 200 mg/kg	Middle cerebral artery occlusion in rats	↓ permeability of BBB ↓ MDA	[116]
Citrus aurantium	50 and 75 mg/kg i.p	Carotid arteries occlusion induced ischemia for 30 min in rats	↓ latency of finding the platform ↑ duration of swimming ↑ anti-oxidant capacity ↓MDA	[117]
Bergamot	0.05, 0.1, 0.5, and 1.0 mL/kg i.p	Middle cerebral artery occlusion induced ischemia in rats	↓infarct size ↓ aspartate and glutamate ↑p-Akt ↑p-GSK-3β	[120]
**Depression**				
Citrus sinensis L. Osbeck and D-limonene	1 mL Citrus sinensis. 50 μL of D-limonene, sniffed	Chronic unpredictable mild stress in mice	↑ weight, sucrose preference ↓ immobility ↓ TG, LDL-C and TC ↑ 5-HT, DA, and NE ↓ CRF and corticosterone ↑ BDNF and Tr-κB	[124]
D-limonene	10 mg/kg orally	Spared nerve injury in rats	↓ immobility	[125]
**Insomnia**				
Anshen	1, 2, 4 × 10^−3^ inhaled	PCPA (300 mg/kg)-induced insomnia in mice	↓ latency of sleeping ↑sleeping time ↑ 5-HT and GABA	[130]
Citrus limon	50, 100 and 150 mg/kg orally	Pentobarbital-induced sleeping time on mice	↑ sleeping time	[77]

Symbol’s indications: ↑; increase, ↓;decrease, (-); reduce, ⇥; activity inhibition.

EOs of natural plants are known to have sedative and hypnotic effects. In addition, aromatherapy is not associated with side effects such as those of traditional psychotropic drugs. Inhaled *Anshen* EOs were assessed by the OFT and pentobarbital-induced sleep time for potential sedative and hypnotic effects. Inhaled *Anshen* EOs consist of seven EOs, including *sandalwood*, *aloe*, *rose*, *lavender*, *frankincense*, *neroli*, and *sweet orange* reduced the spontaneous activity of mice and the latency of sleeping time. It also extended the duration of sleeping time and increased the content of 5-HT and GABA in mice brains [130]. Lopes et al. examined the sedative effects of EOs of leaves from *Citrus limon*, and noticed the same effect as present in the pentobarbital-induced sleeping time test [77]. *Citrus limon* also prolonged the sleeping time period of animals [77].

From a clinical perspective, a randomized, double-blind, placebo-controlled clinical trial was carried out to evaluate the efficiency of *Aloysia citriodora* (*lemon verbena*) in patients suffering from insomnia. The Pittsburgh Sleep Quality Index (PSQI) and Insomnia Severity Index (ISI) questionnaires were used to assess the insomnia severity. Questionnaires were used at three intervals; at the baseline, 2 and 4 weeks after the enrollment. Data analysis showed that scores of global PSQI (measuring sleep latency, habitual sleep efficiency, daytime dysfunction, and subjective sleep quality) and ISI in the *Aloysia citriodora* group were higher after 2 and 4 weeks of treatment when compared with the placebo group [131].

Therefore, the aromatherapy and orally administered EOs containing limonene can be an alternative option for enhancing sleep quality, prolonging duration and preventing insomnia-related complications affecting the daily routine functions of the patients. The pharmacological actions of limonene in sleep disorders, i.e., insomnia, are presented in Figure 5. The available in vivo studies demonstrating the pharmacological mechanisms and actions of limonene in insomnia are presented in Table 3.

## 3. Conclusions

A considerable number of studies revealed a growing interest in using natural limonene to optimize the preventive and therapeutic management of various diseases and strongly supported the hypothesis that limonene is an effective therapeutic agent for neurodegenerative diseases. Limonene is abundant in plant resources, particularly in citrus species, and is widely used in folk medicine that possess medicinal value. Available experimental studies with in vivo, in vitro and epidemiological evidence demonstrate that limonene has the potential to improve age-related neurodegeneration manifested in various diseases, including AD, MS, stroke and others. The available studies are suggestive that limonene and plants or plant products containing limonene may be used to attempt to rationalize their traditional uses or to discover alternative therapies to the current drugs and to reduce adverse effects occurring with the use of current medications. Based on this, it is reasonable that the consumption of a limonene-rich diet as a nutritional agent may contribute in limiting neurodegeneration and aid in preserving cognitive function and maintain neuronal integrity.

## Figures and Tables

**Table 1:** Percentage occurrence of limonene in essential oils.

Plant	Limonene Abundance %
*Citrus medica* L. cv. Diamante peel	15.20
*Piper nigrum*	25.41
*Aloysia citrodora* Palau	13.6–20.1
*Elettaria cardamomum* Seeds	4.35
*Tetraclinis articulata*	7.34
*Cakile maritima* Scop	0.7
*Lippia alba*	6.8
*Pinus roxburghii*	0.9
*Artemisia dracunculus* L.	12.4
*Citrus aurantium* L.	98.66
*Cinnamosma madagascariensis* Danguy	12
*Piper guineense*	5.8
*Terminalia sericea*	0.19
*Pimpinella anisum* L.	1.2
*Citrus latifolia*	58
*Citrus reticulate*	90
*Citrus limon*	52.7
*Citrus sinensis*	97
*Citrus bergamia*	39.6
*Lavandula angustifolia*	19
*Aloysia polystachya*	20.4
*Lavandula angustifolia* Mill	19.6
*Alpinia zerumbet*	8.7
*Nigella sativa*	4.3
*Citrus sinensis* (L.) Osbeck	92.81
*Schinus terebinthifolius*	5–24
Compound Anshen oil *	24.07
*Aloysia citriodora* Palau	6.03

* Compound Anshen consists of seven EOs, including sandalwood essential oil, aloe essential oil, rose essential oil, and lavender essential oil, Frankincense essential oil, neroli essential oil, and sweet orange essential oil.

## Data Availability

Not applicable.

## Sample Availability

Samples of the compound are available from the authors.

## References

[1] Newman D.J. (2008). Natural products as leads to potential drugs: An old process or the new hope for drug discovery?. J. Med. Chem..

[2] Wojtunik-Kulesza K.A., Kasprzak K., Oniszczuk T., Oniszczuk A. (2019). Natural monoterpenes: Much more than only a scent. Chem. Biodivers..

[3] Rodríguez A., Kava V., Latorre-García L., da Silva G.J., Pereira R.G., Glienke C., Ferreira-Maba L.S., Vicent A., Shimada T., Peña L. (2018). Engineering d-limonene synthase down-regulation in orange fruit induces resistance against the fungus phyllosticta citricarpa through enhanced accumulation of monoterpene alcohols and activation of defence. Mol. Plant Pathol..

[4] Murali R., Saravanan R. (2012). Antidiabetic effect of d-limonene, a monoterpene in streptozotocin-induced diabetic rats. Biomed. Prev. Nutr..

[5] Razavi B.M., Arasteh E., Imenshahidi M., Iranshahi M. (2015). Antihypertensive effect of auraptene, a monoterpene coumarin from the genus Citrus, upon chronic administration. Iran. J. Basic Med. Sci..

[6] de Cássia da Silveira e Sá R., Andrade L.N., de Sousa D.P. (2013). A review on anti-inflammatory activity of monoterpenes. Molecules.

[7] Iglesias D.J., Cercós M., Colmenero-Flores J.M., Naranjo M.A., Ríos G., Carrera E., Ruiz-Rivero O., Lliso I., Morillon R., Tadeo F.R. (2007). Physiology of citrus fruiting. Braz. J. Plant Physiol..

[8] Vieira A.J., Beserra F.P., Souza M.C., Totti B.M., Rozza A.L. (2018). Limonene: Aroma of innovation in health and disease. Chem. Biol. Interact..

[9] Kazyoba P., Viljoen A. (2008). Limonene—A review: Biosynthetic, ecological and pharmacological relevance. Nat. Prod. Commun..

[10] Malko M., Wróblewska A. (2016). The importance of R-(+)-limonene as the raw material for organic syntheses and for organic industry. Chemik.

[11] González-Mas M.C., Rambla J.L., López-Gresa M.P., Blázquez M.A., Granell A. (2019). Volatile Compounds in Citrus Essential Oils: A Comprehensive Review. Front. Plant Sci..

[12] Kummer R., Fachini-Queiroz F.C., Estevão-Silva C.F., Grespan R., Silva E.L., Bersani-Amado C.A., Cuman R.K.N. (2013). Evaluation of anti-inflammatory activity of *Citrus latifolia* tanaka essential oil and limonene in experimental mouse models. Evid. Based Complement. Altern. Med..

[13] Bourgou S., Rahali F.Z., Ourghemmi I., Saïdani Tounsi M. (2012). Changes of peel essential oil composition of four tunisian citrus during fruit maturation. Sci. World J..

[14] Ravichandran C., Badgujar P.C., Gundev P., Upadhyay A. (2018). Review of toxicological assessment of d-limonene, a food and cosmetics additive. Food Chem. Toxicol..

[15] Sciacca M.F.M., Romanucci V., Zarrelli A., Monaco I., Lolicato F., Spinella N., Galati C., Grasso G., D’Urso L., Romeo M. (2017). Inhibition of Aβ Amyloid Growth and Toxicity by Silybins: The Crucial Role of Stereochemistry. ACS Chem. Neurosci..

[16] Celia C., Trapasso E., Locatelli M., Navarra M., Ventura C.A., Wolfram J., Carafa M., Morittu V.M., Britti D., Di Marzio L. (2013). Anticancer activity of liposomal bergamot essential oil (BEO) on human neuroblastoma cells. Colloids Surf. B Biointerfaces.

[17] Piccinelli A.C., Morato P.N., Dos Santos Barbosa M., Croda J., Sampson J., Kong X., Konkiewitz E.C., Ziff E.B., Amaya-Farfan J., Kassuya C.A. (2017). Limonene reduces hyperalgesia induced by gp120 and cytokines by modulation of IL-1 β and protein expression in spinal cord of mice. Life Sci..

[18] Moraes T.M., Kushima H., Moleiro F.C., Santos R.C., Rocha L.R., Marques M.O., Vilegas W., Hiruma-Lima C.A. (2009). Effects of limonene and essential oil from Citrus aurantium on gastric mucosa: Role of prostaglandins and gastric mucus secretion. Chem. Biol. Interact..

[19] Roberto D., Micucci P., Sebastian T., Graciela F., Anesini C. (2010). Antioxidant activity of limonene on normal murine lymphocytes: Relation to H_2_O_2_ modulation and cell proliferation. Basic Clin. Pharm. Toxicol..

[20] d’Alessio P.A., Ostan R., Bisson J.F., Schulzke J.D., Ursini M.V., Béné M.C. (2013). Oral administration of d-limonene controls inflammation in rat colitis and displays anti-inflammatory properties as diet supplementation in humans. Life Sci..

[21] Chi G., Wei M., Xie X., Soromou L.W., Liu F., Zhao S. (2013). Suppression of MAPK and NF-κB pathways by limonene contributes to attenuation of lipopolysaccharide-induced inflammatory responses in acute lung injury. Inflammation.

[22] Pohl F., Kong Thoo Lin P. (2018). The potential use of plant natural products and plant extracts with antioxidant properties for the prevention/treatment of neurodegenerative diseases: In vitro, in vivo and clinical trials. Molecules.

[23] Dugger B.N., Dickson D.W. (2017). Pathology of neurodegenerative diseases. Cold Spring Harb. Perspect. Biol..

[24] Velmurugan B., Baskaran R., Bharathi Priya L., Rajan V., Weng C. (2018). Neuroprotective role of phytochemicals. Molecules.

[25] Cutillas A.-B., Carrasco A., Martinez-Gutierrez R., Tomas V., Tudela J. (2018). *Rosmarinus officinalis* L. essential oils from Spain: Composition, antioxidant capacity, lipoxygenase and acetylcholinesterase inhibitory capacities, and antimicrobial activities. Plant Biosyst. Int. J. Deal. Asp. Plant Biol..

[26] https://www.x-mol.com/paper/1337142821747023872.

[27] Nussbaum R.L., Ellis C.E. (2003). Alzheimer’s disease and Parkinson’s disease. N. Engl. J. Med..

[28] Szwajgier D., Baranowska-Wójcik E. (2019). Terpenes and phenylpropanoids as acetyl- and butyrylcholinesterase inhibitors: A comparative study. Curr. Alzheimer Res..

[29] Tramutola A., Triani F., Di Domenico F., Barone E., Cai J., Klein J.B., Perluigi M., Butterfield D.A. (2018). Poly-ubiquitin profile in Alzheimer disease brain. Neurobiol. Dis..

[30] Lenz S., Karsten P., Schulz J.B., Voigt A. (2013). Drosophila as a screening tool to study human neurodegenerative diseases. J. Neurochem..

[31] Shin M., Liu Q.F., Choi B., Shin C., Lee B., Yuan C., Song Y.J., Yun H.S., Lee I.S., Koo B.S. (2020). Neuroprotective effects of limonene (+) against Aβ42-induced neurotoxicity in a drosophila model of Alzheimer’s disease. Biol. Pharm Bull.

[32] Sánchez-Martínez J.D., Bueno M., Alvarez-Rivera G., Tudela J., Ibañez E., Cifuentes A. (2021). In vitro neuroprotective potential of terpenes from industrial orange juice by-products. Food Funct..

[33] Conforti F., Statti G.A., Tundis R., Loizzo M.R., Menichini F. (2007). In vitro activities of *Citrus medica* L. cv. Diamante (Diamante citron) relevant to treatment of diabetes and Alzheimer’s disease. Phytother. Res..

[34] Boiangiu R.S., Brinza I., Hancianu M., Erdogan Orhan I., Eren G., Gündüz E., Ertas H., Hritcu L., Cioanca O. (2020). Cognitive facilitation and antioxidant effects of an essential oil mix on scopolamine-induced amnesia in rats: Molecular modeling of in vitro and in vivo approaches. Molecules.

[35] Zhou W., Fukumoto S., Yokogoshi H. (2009). Components of lemon essential oil attenuate dementia induced by scopolamine. Nutr. Neurosci..

[36] Sadiki F.Z., Idrissi M.E., Cioanca O., Trifan A., Hancianu M., Hritcu L., Postu P.A. (2019). Tetraclinis articulata essential oil mitigates cognitive deficits and brain oxidative stress in an Alzheimer’s disease amyloidosis model. Phytomedicine.

[37] Abuhamdah S., Abuhamdah R., Howes M.J., Al-Olimat S., Ennaceur A., Chazot P.L. (2015). Pharmacological and neuroprotective profile of an essential oil derived from leaves of *Aloysia citrodora* Palau. J. Pharm Pharm..

[38] Lomarat P., Sripha K., Phanthong P., Kitphati W., Thirapanmethee K., Bunyapraphatsara N. (2015). In vitro biological activities of black pepper essential oil and its major components relevant to the prevention of Alzheimer’s disease. Thai J. Pharm. Sci. (TJPS).

[39] Zéphir H. (2018). Progress in understanding the pathophysiology of multiple sclerosis. Rev. Neurol..

[40] Elshamy A. (2016). Cakile maritima Scop. extracts inhibit the growth of some bacterial triggers of autoimmune diseases: GC-MS analysis of an inhibitory extract. Pharmacogn. J..

[41] Nel A.L., Murhekar S., Matthews B., White A., Cock I.E. (2020). The interactive antimicrobial activity of Terminalia sericea Burch ex DC. leaf extracts and conventional antibiotics against bacterial triggers of selected autoimmune inflammatory diseases. S. Afr. J. Bot..

[42] Maayah Z.H., Takahara S., Ferdaoussi M., Dyck J.R.B. (2020). The molecular mechanisms that underpin the biological benefits of full-spectrum cannabis extract in the treatment of neuropathic pain and inflammation. Biochim. Biophys. Acta (BBA) Mol. Basis Dis..

[43] Ben-Shabat S., Fride E., Sheskin T., Tamiri T., Rhee M.H., Vogel Z., Bisogno T., De Petrocellis L., Di Marzo V., Mechoulam R. (1998). An entourage effect: Inactive endogenous fatty acid glycerol esters enhance 2-arachidonoyl-glycerol cannabinoid activity. Eur. J. Pharm..

[44] Leussink V.I., Husseini L., Warnke C., Broussalis E., Hartung H.P., Kieseier B.C. (2012). Symptomatic therapy in multiple sclerosis: The role of cannabinoids in treating spasticity. Ther. Adv. Neurol Disord..

[45] Ku C.-M., Lin J.-Y. (2013). Anti-inflammatory effects of 27 selected terpenoid compounds tested through modulating Th1/Th2 cytokine secretion profiles using murine primary splenocytes. Food Chem..

[46] de Almeida A.A.C., Silva R.O., Nicolau L.A.D., de Brito T.V., de Sousa D.P., Barbosa A.L.d.R., de Freitas R.M., Lopes L.d.S., Medeiros J.-V.R., Ferreira P.M.P. (2017). Physio-pharmacological investigations about the anti-inflammatory and antinociceptive efficacy of (+)-limonene epoxide. Inflammation.

[47] Pietrobon D., Moskowitz M. (2012). Pathophysiology of migraine. Annu. Rev. Physiol..

[48] Mosavat S.H., Jaberi A.R., Sobhani Z., Mosaffa-Jahromi M., Iraji A., Moayedfard A. (2019). Efficacy of Anise (*Pimpinella anisum* L.) oil for migraine headache: A pilot randomized placebo-controlled clinical trial. J. Ethnopharmacol..

[49] Conde R., Corrêa V.S., Carmona F., Contini S.H., Pereira A.M. (2011). Chemical composition and therapeutic effects of Lippia alba (Mill.) N. E. Brown leaves hydro-alcoholic extract in patients with migraine. Phytomedicine.

[50] Baron E.P., Lucas P., Eades J., Hogue O. (2018). Patterns of medicinal cannabis use, strain analysis, and substitution effect among patients with migraine, headache, arthritis, and chronic pain in a medicinal cannabis cohort. J. Headache Pain.

[51] Giourou E., Stavropoulou-Deli A., Giannakopouou A., Kostopoulos G., Koutroumanidis M. (2015). Introduction to Epilepsy and Related Brain Disorders. Cyberphysical Systems for Epilepsy and Related Brain Disorders.

[52] Falco-Walter J.J., Scheffer I.E., Fisher R.S. (2018). The new definition and classification of seizures and epilepsy. Epilepsy Res..

[53] Johannessen Landmark C., Patsalos P.N. (2010). Drug interactions involving the new second- and third-generation antiepileptic drugs. Expert Rev. Neurother..

[54] Sayyah M., Nadjafnia L., Kamalinejad M. (2004). Anticonvulsant activity and chemical composition of *Artemisia dracunculus* L. essential oil. J. Ethnopharmacol..

[55] Azanchi T., Shafaroodi H., Asgarpanah J. (2014). Anticonvulsant activity of Citrus aurantium blossom essential oil (neroli): Involvment of the GABAergic system. Nat. Prod. Commun..

[56] Abbasnejad M., Keramat B., Esmaeili Mahani S., Rezaeezade-Roukerd M. (2012). Effect of hydro-methanolic extract of sour orange flowers, citrus aurantium, on pentylentetrazole induced seizure in male rats. J. Babol Univ. Med. Sci..

[57] Rosa-Falero C., Torres-Rodríguez S., Jordán C., Licier R., Santiago Y., Toledo Z., Santiago M., Serrano K., Sosa J., Ortiz J.G. (2015). Citrus aurantium increases seizure latency to PTZ induced seizures in zebrafish thru NMDA and mGluR’s I and II. Front. Pharmacol..

[58] Viana G.S., do Vale T.G., Silva C.M., Matos F.J. (2000). Anticonvulsant activity of essential oils and active principles from chemotypes of Lippia alba (Mill.) N.E. Brown. Biol. Pharm. Bull..

[59] Rakotosaona R., Randrianarivo E., Rasoanaivo P., Nicoletti M., Benelli G., Maggi F. (2017). Effect of the Leaf Essential Oil from Cinnamosma madagascariensis Danguy on Pentylenetetrazol-induced Seizure in Rats. Chem. Biodivers..

[60] Oyemitan I.A., Olayera O.A., Alabi A., Abass L.A., Elusiyan C.A., Oyedeji A.O., Akanmu M.A. (2015). Psychoneuropharmacological activities and chemical composition of essential oil of fresh fruits of Piper guineense (Piperaceae) in mice. J. Ethnopharmacol..

[61] Pathak S., Wanjari M.M., Jain S.K., Tripathi M. (2010). Evaluation of Antiseizure Activity of Essential Oil from Roots of Angelica archangelica Linn. in Mice. Indian J. Pharm. Sci..

[62] Alam O., Mullick P., Verma S.P., Gilani S.J., Khan S.A., Siddiqui N., Ahsan W. (2010). Synthesis, anticonvulsant and toxicity screening of newer pyrimidine semicarbazone derivatives. Eur. J. Med. Chem..

[63] Rajak H., Singh Thakur B., Singh A., Raghuvanshi K., Sah A.K., Veerasamy R., Sharma P.C., Singh Pawar R., Kharya M.D. (2013). Novel limonene and citral based 2,5-disubstituted-1,3,4-oxadiazoles: A natural product coupled approach to semicarbazones for antiepileptic activity. Bioorg. Med. Chem. Lett..

[64] Cannistraro P.A., Rauch S.L. (2003). Neural circuitry of anxiety: Evidence from structural and functional neuroimaging studies. Psychopharmacol. Bull..

[65] Adwas A., Jbireal J., Azab A. (2019). Anxiety: Insights into signs, symptoms, etiology, pathophysiology, and treatment. S. Afr. J. Med. Sci..

[66] Nemeroff C.B. (2003). The role of GABA in the pathophysiology and treatment of anxiety disorders. Psychopharmacol. Bull..

[67] Ressler K.J., Nemeroff C.B. (2000). Role of serotonergic and noradrenergic systems in the pathophysiology of depression and anxiety disorders. Depress. Anxiety.

[68] Nash J.R., Nutt D.J. (2005). Pharmacotherapy of anxiety. Handb. Exp. Pharm..

[69] Lydiard R.B. (2003). The role of GABA in anxiety disorders. J. Clin. Psychiatry.

[70] Khakpour S., Khosravi M., Mashayekhipour Z., Hadipour Jahromy M. (2014). Effect of *Citrus aurantium* L. essential oil and haloperidol on anxiety in male mice. World J. Neurosci..

[71] Khosravi M., Khakpour S., Adibi L., Hadipour Jahromy M. (2014). A Study of the effect of *Citrus aurantium* L. essential oil on anxiety and its interaction with gabaergic pathways in male mice. J. Behav. Brain Sci..

[72] Pultrini Ade M., Galindo L.A., Costa M. (2006). Effects of the essential oil from *Citrus aurantium* L. in experimental anxiety models in mice. Life Sci..

[73] Silva Brum L.F., Emanuelli T., Souza D.O., Elisabetsky E. (2001). Effects of linalool on glutamate release and uptake in mouse cortical synaptosomes. Neurochem. Res..

[74] Carvalho-Freitas M.I., Costa M. (2002). Anxiolytic and sedative effects of extracts and essential oil from *Citrus aurantium* L.. Biol. Pharm. Bull..

[75] Costa C., Cury T., Cassettari B., Takahira R., Flório J., Costa M. (2013). *Citrus aurantium* L. essential oil exhibits anxiolytic-like activity mediated by 5-HT1A-receptors and reduces cholesterol after repeated oral treatment. BMC Complement. Altern. Med..

[76] Gargano A. (2008). Essential oils from citrus latifolia and citrus reticulata reduce anxiety and prolong ether sleeping time in mice. Tree For. Sci. Biotechnol..

[77] Lopes Campêlo L.M., Gonçalves e Sá C., de Almeida A.A., da Costa J.P., Marques T.H., Feitosa C.M., Saldanha G.B., de Freitas R.M. (2011). Sedative, anxiolytic and antidepressant activities of Citrus limon (Burn) essential oil in mice. Pharmazie.

[78] Faturi C.B., Leite J.R., Alves P.B., Canton A.C., Teixeira-Silva F. (2010). Anxiolytic-like effect of sweet orange aroma in Wistar rats. Prog. Neuropsychopharmacol. Biol. Psychiatry.

[79] Mesfin M., Asres K., Shibeshi W. (2014). Evaluation of anxiolytic activity of the essential oil of the aerial part of Foeniculum vulgare Miller in mice. BMC Complement. Altern. Med..

[80] Saiyudthong S., Marsden C.A. (2011). Acute effects of bergamot oil on anxiety-related behaviour and corticosterone level in rats. Phytother. Res..

[81] Morrone L.A., Rombolà L., Pelle C., Corasaniti M.T., Zappettini S., Paudice P., Bonanno G., Bagetta G. (2007). The essential oil of bergamot enhances the levels of amino acid neurotransmitters in the hippocampus of rat: Implication of monoterpene hydrocarbons. Pharm. Res..

[82] Bartanusz V., Muller D., Gaillard R.C., Streit P., Vutskits L., Kiss J.Z. (2004). Local gamma-aminobutyric acid and glutamate circuit control of hypophyseotrophic corticotropin-releasing factor neuron activity in the paraventricular nucleus of the hypothalamus. Eur. J. Neurosci..

[83] Zhou W., Yoshioka M., Yokogoshi H. (2009). Sub-Chronic Effects of s-Limonene on Brain Neurotransmitter Levels and Behavior of Rats. J. Nutr. Sci. Vitaminol..

[84] Rombolà L., Tridico L., Scuteri D., Sakurada T., Sakurada S., Mizoguchi H., Avato P., Corasaniti M.T., Bagetta G., Morrone L.A. (2017). Bergamot Essential Oil Attenuates Anxiety-Like Behaviour in Rats. Molecules.

[85] Jafarzadeh M., Arman S., Pour F.F. (2013). Effect of aromatherapy with orange essential oil on salivary cortisol and pulse rate in children during dental treatment: A randomized controlled clinical trial. Adv. Biomed. Res..

[86] Lehrner J., Eckersberger C., Walla P., Pötsch G., Deecke L. (2000). Ambient odor of orange in a dental office reduces anxiety and improves mood in female patients. Physiol. Behav..

[87] Lehrner J., Marwinski G., Lehr S., Johren P., Deecke L. (2005). Ambient odors of orange and lavender reduce anxiety and improve mood in a dental office. Physiol. Behav..

[88] Carmona F., Coneglian F.S., Batista P.A., Aragon D.C., Angelucci M.A., Martinez E.Z., Pereira A.M.S. (2019). Aloysia polystachya (Griseb.) Moldenke (Verbenaceae) powdered leaves are effective in treating anxiety symptoms: A phase-2, randomized, placebo-controlled clinical trial. J. Ethnopharmacol..

[89] Soto Vásquez M.R., Alvarado García P. (2018). Anxiolytic-like effect of Lippia alba essential oil: A randomized, placebo-controlled trial. J. Complementary Med. Res..

[90] Goes T.C., Antunes F.D., Alves P.B., Teixeira-Silva F. (2012). Effect of sweet orange aroma on experimental anxiety in humans. J. Altern. Complement. Med..

[91] Bakhsha F., Mazandarani M., Aryaei M., Jafari S., Bayate H. (2014). Phytochemical and anti-oxidant activity of lavandula angustifolia mill. essential oil on preoperative anxiety in patients undergoing diagnostic curettage. Int. J. Women’s Health Reprod. Sci..

[92] Braden R., Reichow S., Halm M.A. (2009). The use of the essential oil lavandin to reduce preoperative anxiety in surgical patients. J. Perianesthesia Nurs..

[93] Tsigos C., Chrousos G.P. (2002). Hypothalamic-pituitary-adrenal axis, neuroendocrine factors and stress. J. Psychosom Res..

[94] d’Alessio P.A., Bisson J.F., Béné M.C. (2014). Anti-stress effects of d-limonene and its metabolite perillyl alcohol. Rejuvenation Res..

[95] Salminen A., Hyttinen J.M., Kaarniranta K. (2011). AMP-activated protein kinase inhibits NF-κB signaling and inflammation: Impact on healthspan and lifespan. J. Mol. Med..

[96] Noort A.R., van Zoest K.P.M., Weijers E.M., Koolwijk P., Maracle C.X., Novack D.V., Siemerink M.J., Schlingemann R.O., Tak P.P., Tas S.W. (2014). NF-κB-inducing kinase is a key regulator of inflammation-induced and tumour-associated angiogenesis. J. Pathol..

[97] Tang X.P., Guo X.H., Geng D., Weng L.J. (2019). d-Limonene protects PC12 cells against corticosterone-induced neurotoxicity by activating the AMPK pathway. Environ. Toxicol. Pharm..

[98] Lima N.G., De Sousa D.P., Pimenta F.C., Alves M.F., De Souza F.S., Macedo R.O., Cardoso R.B., de Morais L.C., Melo Diniz Mde F., de Almeida R.N. (2013). Anxiolytic-like activity and GC-MS analysis of (R)-(+)-limonene fragrance, a natural compound found in foods and plants. Pharm. Biochem. Behav..

[99] Bigdeli Y., Asle-Rousta M., Rahnema M. (2019). Effects of limonene on chronic restraint stress-induced memory impairment and anxiety in male rats. Neurophysiology.

[100] de Almeida A.A., Costa J.P., de Carvalho R.B., de Sousa D.P., de Freitas R.M. (2012). Evaluation of acute toxicity of a natural compound (+)-limonene epoxide and its anxiolytic-like action. Brain Res..

[101] de Almeida A.A.C., de Carvalho R.B.F., Silva O.A., de Sousa D.P., de Freitas R.M. (2014). Potential antioxidant and anxiolytic effects of (+)-limonene epoxide in mice after marble-burying test. Pharmacol. Biochem. Behav..

[102] Satou T., Murakami S., Matsuura M., Hayashi S., Koike K. (2010). Anxiolytic effect and tissue distribution of inhaled Alpinia zerumbet essential oil in mice. Nat. Prod. Commun..

[103] Murakami S., Matsuura M., Satou T., Hayashi S., Koike K. (2009). Effects of the essential oil from leaves of Alpinia zerumbet on behavioral alterations in mice. Nat. Prod. Commun..

[104] Chugh C. (2019). Acute Ischemic Stroke: Management Approach. Indian J. Crit. Care Med. Peer-Rev. Off. Publ. Indian Soc. Crit. Care Med..

[105] Shalak L., Perlman J.M. (2004). Hypoxic–ischemic brain injury in the term infant-current concepts. Early Hum. Dev..

[106] Hill M.D. (2008). What kind of stroke is it?. Clin. Chem..

[107] Muir K. (2001). Medical management of Stroke. J. Neurol. Neurosurg. Psychiatry.

[108] Bayazit V. (2010). Assessment of effects of nanomaterials in fennel (Foeniculum vulgare miller) seed on the cloth dissolution after spontaneously stroke of male mole rat (Spalax leucodon) in Muş, Turkey. Dig. J. Nanomater. Biostruct..

[109] Wang X., Li G., Shen W. (2018). Protective effects of D-Limonene against transient cerebral ischemia in stroke-prone spontaneously hypertensive rats. Exp. Ther. Med..

[110] Rosand J., Schwamm L.H. (2001). Management of Brain Edema Complicating Stroke. J. Intensive Care Med..

[111] Vakili A., Sharifat S., Akhavan M.M., Bandegi A.R. (2014). Effect of lavender oil (*Lavandula angustifolia*) on cerebral edema and its possible mechanisms in an experimental model of stroke. Brain Res..

[112] Rabiei Z., Bigdeli M.R., Rasoulian B., Ghassempour A., Mirzajani F. (2012). The neuroprotection effect of pretreatment with olive leaf extract on brain lipidomics in rat stroke model. Phytomed. Int. J. Phytother. Phytopharm..

[113] Ali B.H., Blunden G. (2003). Pharmacological and toxicological properties of Nigella sativa. Phytother. Res..

[114] Hosseinzadeh H., Parvardeh S., Asl M.N., Sadeghnia H.R., Ziaee T. (2007). Effect of thymoquinone and Nigella sativa seeds oil on lipid peroxidation level during global cerebral ischemia-reperfusion injury in rat hippocampus. Phytomedicine.

[115] Gasche Y., Copin J.C., Sugawara T., Fujimura M., Chan P.H. (2001). Matrix metalloproteinase inhibition prevents oxidative stress-associated blood-brain barrier disruption after transient focal cerebral ischemia. J. Cereb. Blood Flow Metab..

[116] Rabiei Z., Rafieian-Kopaei M. (2014). Neuroprotective effect of pretreatment with Lavandula officinalis ethanolic extract on blood-brain barrier permeability in a rat stroke model. Asian Pac. J. Trop. Med..

[117] Sadeghimanesh A., Khalaji-Pirbalouty V., Lorigooini Z., Rafieian-kopaei M., Torki A., Rabiei Z. (2018). Phytochemical and neuroprotective evaluation of Citrus aurantium essential oil on cerebral ischemia and reperfusion. Bangladesh J. Pharmacol..

[118] Zhao H., Sapolsky R.M., Steinberg G.K. (2006). Phosphoinositide-3-kinase/akt survival signal pathways are implicated in neuronal survival after stroke. Mol. Neurobiol..

[119] Corasaniti M.T., Maiuolo J., Maida S., Fratto V., Navarra M., Russo R., Amantea D., Morrone L.A., Bagetta G. (2007). Cell signaling pathways in the mechanisms of neuroprotection afforded by bergamot essential oil against NMDA-induced cell death in vitro. Br. J. Pharm..

[120] Amantea D., Fratto V., Maida S., Rotiroti D., Ragusa S., Nappi G., Bagetta G., Corasaniti M.T. (2009). Prevention of glutamate accumulation and upregulation of phospho-akt may account for neuroprotection afforded by bergamot essential oil against brain injury induced by focal cerebral ischemia in rat. Int. Rev. Neurobiol..

[121] Vasilopoulou C., Bourtsi E., Giaple S., Koutelekos I., Theofilou P., Polikandrioti M. (2015). The Impact of Anxiety and Depression on the Quality of Life of Hemodialysis Patients. Glob. J. Health Sci..

[122] Kennedy S.H., Andersen H.F., Thase M.E. (2009). Escitalopram in the treatment of major depressive disorder: A meta-analysis. Curr. Med. Res. Opin.

[123] Satou T., Hayakawa M., Kasuya H., Masuo Y., Koike K. (2017). Mouse brain concentrations of α-pinene, limonene, linalool, and 1,8-cineole following inhalation. Flavour Fragr. J..

[124] Zhang L.L., Yang Z.Y., Fan G., Ren J.N., Yin K.J., Pan S.Y. (2019). Antidepressant-like effect of *Citrus sinensis* (L.) osbeck essential oil and its main component limonene on mice. J. Agric. Food Chem..

[125] Piccinelli A.C., Santos J.A., Konkiewitz E.C., Oesterreich S.A., Formagio A.S., Croda J., Ziff E.B., Kassuya C.A. (2015). Antihyperalgesic and antidepressive actions of (R)-(+)-limonene, α-phellandrene, and essential oil from Schinus terebinthifolius fruits in a neuropathic pain model. Nutr. Neurosci..

[126] Chaiyasut C., Sivamaruthi B.S., Wongwan J., Thiwan K., Rungseevijitprapa W., Klunklin A., Kunaviktikul W. (2020). Effects of *Litsea cubeba* (Lour.) persoon essential oil aromatherapy on mood states and salivary cortisol levels in healthy volunteers. Evid. Based Complement. Altern. Med..

[127] Roth T. (2007). Insomnia: Definition, prevalence, etiology, and consequences. J. Clin. Sleep Med. JCSM Off. Publ. Am. Acad. Sleep Med..

[128] Krystal A.D., Prather A.A., Ashbrook L.H. (2019). The assessment and management of insomnia: An update. World Psychiatry Off. J. World Psychiatr. Assoc. (WPA).

[129] Sieghart W., Sperk G. (2002). Subunit composition, distribution and function of GABA(A) receptor subtypes. Curr. Top Med. Chem..

[130] Zhong Y., Zheng Q., Hu P., Huang X., Yang M., Ren G., Du Q., Luo J., Zhang K., Li J. (2019). Sedative and hypnotic effects of compound Anshen essential oil inhalation for insomnia. BMC Complement. Altern. Med..

[131] Afrasiabian F., Mirabzadeh Ardakani M., Rahmani K., Azadi N.A., Alemohammad Z.B., Bidaki R., Karimi M., Emtiazy M., Hashempur M.H. (2019). Aloysia citriodora Palau (lemon verbena) for insomnia patients: A randomized, double-blind, placebo-controlled clinical trial of efficacy and safety. Phytother. Res..

